# Reasons why insured consumers co-pay for medicines at retail pharmacies in Pretoria, South Africa

**DOI:** 10.4102/phcfm.v11i1.1878

**Published:** 2019-03-27

**Authors:** Ntobeko M. Mpanza, Hazel Bradley, Richard Laing

**Affiliations:** 1 School of Public Health, University of the Western Cape, Bellville, South Africa; 2 Department of Global Health, School of Public Health, Boston University, Massachusetts, United States

## Abstract

**Background:**

Costly prescription medicines with existing cheaper alternatives tend to be purchased by medically insured consumers of healthcare. In South Africa medical scheme members pay higher out-of-pocket payments for medicines than those without insurance.

**Aim:**

This study explored reasons for co-payments among insured Pretoria medical scheme members purchasing prescription medicines at private retail pharmacies, despite being insured and protected against such payments.

**Setting:**

The study took place in retail pharmacies in Pretoria, Gauteng Province, South Africa.

**Methods:**

An exploratory qualitative study was performed. Semi-structured interviews were conducted among purposefully sampled medical scheme members (12) and nine key informants (six pharmacists and three regulators – one for the pharmaceutical industry, one for medical schemes and one for pharmacists). Three pharmacies (two corporate and one independent) each were identified from high and low socio-economic areas. Scheme members were interviewed immediately after having made a co-payment (eight) or no co-payment (four) from the selected pharmacies. Interviews were recorded, coded and organised into themes.

**Results:**

Co-payments were deemed confusing, unpredictable and inconsistent between and within pharmacies. Members blamed schemes for causing co-payments. Six sampled pharmacies rarely stocked the lowest-priced medicines; instead, they dispensed medicines from manufacturers with whom they had a relationship. Corporate pharmacies were favoured compared to independents and brand loyalty superseded cost considerations. Medical scheme members did not understand how medical schemes’ function.

**Conclusion:**

Unavailability of lowest-priced medicines at pharmacies contributes to co-payments. Consumer education about generics and expedited implementation of National Health Insurance could significantly reduce co-payments.

**Keywords:**

co-payments; high socio-economic; low socio-economic; medical scheme; medicines pricing policy; National Health Insurance; pharmacist; prescribed minimum benefit; retail pharmacy; regulator.

## Introduction

In the United States (US), high healthcare expenditure is observed in private sector settings and rapid growth in cost is observed in prescription medicines and administrative costs of private health insurance.^1^ Similarly, in South Africa, insured medical scheme members incur high co-payments for services offered in private sector facilities.^2,3^ Existing and proposed legislation in the country, such as the *Medicines and Related Substances Act 101 of 1965, Pharmacy Act 53 of 1974, Medical Schemes Act 131 of 1998* and National Health Insurance Policy, collectively aim to offer consumers transparency in the pricing of medicines, the right of access to cheaper prescription medicines and protection from financial ruin because of healthcare costs.^4,5,6,7^ Realisation of positive outcomes intended by introducing these policies is only possible if consumers are fully informed about available options, to minimise co-payments, by health professionals responsible for the prescribing and dispensing of medicines.

The *Medical Schemes Act*, for example, compels medical schemes to offer full reimbursement for formulary medicines used to treat medical conditions listed on the prescribed minimum benefit (PMB) schedule.^8^ However, the benefit packages of medical scheme organisations differ, thus making it necessary for each registered member to understand the terms and conditions of coverage offered to them. To qualify for full financial coverage members are expected to purchase medicines that appear on the formulary list; otherwise they incur co-payments. Those not adhering to this requirement become liable for additional costs such as co-payments, especially if services rendered cost more than the scheme’s reimbursement threshold.^8^ Other medical schemes allocate funds into their member’s savings account to cater for costs such as physician consultations and medicines. These funds represent a member’s share of the maximum allowable expenditure towards costs such as prescription medicines. When funds are depleted, the member pays cash out-of-pocket (OOP) and this happens even when membership monthly contributions continue to be debited by the medical scheme.^8^ Generally, health insurance organisations introduce co-payments and formularies as measures aimed at controlling high expenditure on prescription medicines; however, in the USA, some members enrolled in these medical schemes believe that these measures are designed to save money for health insurance companies and their subsidiaries.^9,10,11^

In 2015, the annual average increase in member contributions in South Africa was reported to be 9.2%, almost double Consumer Price Index (CPI) (4.6%) at the time.^12^ Harris et al.^13^ suggested that being medically insured in South Africa is linked to financial ruin. For example, it was reported that in 2005 spending per private medical scheme member was ninefold higher than public sector expenditure.^3,14^ Ataguba et al.^15^ conducted a study in South Africa to assess the extent to which medical scheme membership led to an increase or reduction in OOP payments. The study concluded that medical scheme members paid higher healthcare charges than non-members and proposed the speedy implementation of the planned National Health Insurance.^15^ However, the study did not explore factors affecting co-payments from the perspective of insured medical scheme members. The aim of this study was therefore to explore views about co-payments and identify factors that influenced Pretoria medical scheme members to co-pay when purchasing prescription medicines at pharmacies, despite already being insured by medical scheme insurance organisations.

## Methods

An exploratory qualitative study was conducted at community pharmacies in South Africa’s capital city, Pretoria. A purposive sample comprising 12 medical scheme members and nine key informants (KIs) was selected (Table 1). The KIs included six pharmacists and three regulators – one for pharmacists, one for medical schemes and one for the pharmaceutical industry. One of the regulators had extensive experience both as a pharmaceutical industry regulator and a representative of medical schemes. Medical scheme members and pharmacists were interviewed at the premises of six purposively sampled private community pharmacies – three pharmacies each (two corporate and one independent) were identified from high and low socio-economic areas; and the other KIs were interviewed at their workplaces. Medical scheme members provided insights and understanding of factors that have an influence on co-payments for medicines from their personal experiences. The role of KIs was to provide views about co-payments in relation to existing medicine policies, dispensing practices and any known consumer-related experiences at South African private sector retail pharmacies. The interviews of medical scheme members were much shorter (10–30 min) than those of KIs (46–90 min). Interviews were recorded using a recording device and interview notes were captured in a diary during, and immediately after, each interview. Codes identified from recordings and interview notes were transformed into organised text for theme development.

**TABLE 1:** Profile of interviewees and interview locations.

Variable	High socio-economic location	Low socio-economic location
**A: Medical scheme members (12)**		
Corporate pharmacies	KER_M12 Co-payer	LES_M11 Co-payer
	NEL_M4 Co-payer	EST_M10 Co-payer
	DUM_M8 Non-co-payer	MAL_ Non-co-payer
Independent pharmacies	BAB_M9 Co-payer	MOR_M5 Co-payer
	FUT_M3 Co-payer	DIR_ M1 Co-payer
	MAN_M6 Non-co-payer	FRI_M7 Non-co-payer
**B: Pharmacists (6)**		
Corporate pharmacies	VER_P6 Pharmacist	SUN_P3 Pharmacist
	DIS_P2 Pharmacist	KAL_P4 Pharmacist
Independent pharmacies	CRO_P5 Pharmacist	KEM_P1 Pharmacist
**C: Regulators (3)**		
Regulator 1	Pharmacists’ regulator	
Regulator 2	Medical schemes’ regulator	
Regulator 3	Medical schemes’ representative and former regulator of medicines	

### Ethical considerations

Ethical clearance was granted by the University of the Western Cape Senate Research Committee (Registration No. 15/7/8).

## Results

Ten themes were identified to represent interviewee views about co-payments, as well as purchasing behaviour and perceptions that result in OOP payments, and they are presented in the following section.

### Feelings and views about co-payments

Pharmacists and medical scheme members were confused by the unpredictability and inconsistencies in co-payment charges:

‘I noticed that prices are different at different pharmacies. You’d find the pharmacy doesn’t charge you, but next time you buy the same Demazin you pay an extra R16.00. The confusing thing is, they never asked me to pay the last time, I don’t understand that.’ (DIR_M1, female, low-income independent co-payer)

‘I am not happy about co-payments, but also you want the business to be profitable. I can’t say I know how the co-payment is calculated. We don’t have information on these things. Medical schemes decrease their price but our price continues to increase and the patient co-pays. You find a medicine that didn’t require co-payment suddenly requires a co-payment a month later, I fail to understand this.’ (VER_P6, male, pharmacist at a corporate pharmacy located in a high-income location)

The determination of co-payment amounts appears to be decided elsewhere, outside of the control of dispensing pharmacists, and the amounts charged at pharmacies were not transparent to the medical scheme members.

### Co-payment perceptions between co-payers and non-co-payers

The non-co-payers were equally confused because they lacked understanding about reasons why they did not co-pay. Some blamed the medical scheme and they believed that relief from payment, if it happened, was only for a short while:

‘I hardly experience a co-payment because I belong to a very tricky medical scheme. They pay from my savings account. This allows you to choose either generic or original and once your savings are up [*finished*] then you might need to co-pay because there is an amount that is outstanding.’ (MAN_M6, female, high-income independent non-co-payer)

‘I have never co-paid for my chronic medicines. Maybe because I only go to this one doctor that I was told to use by my medical aid. I usually leave my prescription at the pharmacy and come back later to collect my medication without paying anything.’ (MAL_M2, male, low-income corporate non-co-payer)

The complicated and dynamic nature in which medical schemes offer packages poses a challenge to members’ and/or pharmacists’ understanding of the scheme’s structure.

### Co-payment views from members of low versus high socio-economic areas

To low-income medical scheme members, co-payments were considered a financial matter, whereas high-income members were frustrated by the incomprehensible co-payment information provided to them by medical schemes:

‘I always have to co-pay and I don’t even know why I must pay extra. The money was too much and I can’t afford it. I even went to the schemes offices to find out why I pay so much extra money, but they are useless. Remember, I already pay monthly and again I have to pay at pharmacy X.’ (MOR_M5, female, low-income independent co-payer)

‘… every year they come and try to explain about these co-payments. Sometimes you don’t even understand because what they say becomes so complicated. They say you haven’t reached the gap and the threshold and all those things.’ (FUT_ M3, female, high-income independent co-payer)

The members expected more benefits compared to what medical schemes offered to them. Medical schemes tended to be proactive in offering information to high socio-economic area medical scheme members, whereas members from low socio-economic areas were more likely to approach the scheme for clarity.

### Lack of understanding of how medical schemes function

The terminology used by medical schemes appeared to contribute to the inability for members to understand how schemes work:

‘You just listen but you don’t understand what is going on. Even today I don’t even know what’s the threshold, when do I get into that threshold, when do I come out of the threshold, and when that threshold is over then how much do they pay.’ (FUT_ M3, female, high-income independent co-payer)

The medical scheme members seemed to be intimidated by the terminology used by schemes and this resulted in the members’ lack of interest in addressing financial matters with schemes.

### The perceived role of medical schemes in causing co-payments

Key informants justified co-payments from the perspective of medical schemes, who were considered victims to high claims from service providers. Co-payments were explained as a mechanism used by medical schemes to share high healthcare costs with members. The alignment of the schemes’ businesses with the hospicentric health model and the schemes’ interests in non-healthcare activities were regarded as contributing factors to high costs experienced by members:

‘… medical schemes behave in a manner that gives them a bad name because they are faced with huge payments on the claims side … and services are not properly regulated … service providers charge whatever they feel like, without adhering to any benchmark. What schemes end up doing then is to try and find ways and means of decreasing what gets paid out.’ (Regulator 3, male, doctor)

‘… Medical aid scheme benefits to members are structured around PMBs. PMBs cater for more hospicentric specialist centric type of benefits. So schemes don’t have the incentive to have normal primary and preventative healthcare benefits for day-to-day costs. Most of them that do have it use funds from savings accounts; that’s where most of your day-to-day benefits are funded from for the majority … of the schemes.’ (Regulator 2, male, economist)

‘You hear members say they’ve run out of benefits round about September; the next day you hear that medical schemes have sponsored a soccer team, a marathon, a bike race etc., etc., with members’ money, then you become concerned about what exactly schemes are doing at the expense of the members? The question is, is the scheme having the member’s interests at heart or there are other things that are happening with the member’s money that the people running the scheme benefit from, not the member? The medical aid schemes do this, knowing that for an ordinary person it would be very difficult to opt out because of the emotional linkage of the member to the scheme.’ (Regulator 3, male, doctor)

Data suggested that medical schemes have other financial interests that are fulfilled using membership contributions. Scheme membership, on the other hand, is viewed as an emotional enrolment caused by the member’s concern for their health and that of family members.

### The influence of the state of ill-health

Most members stated that when presenting prescriptions at pharmacies they focused on getting better and were less interested in discussions that had the potential to exacerbate the condition that brought them to the pharmacy in the first place:

‘What if you ask and you find that you don’t understand what they are telling you when they explain why you are co-paying? [*pause*] Remember you are already upset because you are sick and because you are sick you don’t want to be more upset. So … you … just pay … and get your medicines and you don’t want to ask about it.’ (NEL_ M4, female, high-income corporate co-payer)

‘When you are sick you are not thinking about money. All you need is to get better and anything that makes you want to argue, like co-payments, will upset you even more than you are already. Your state of mind is such that you just want to get your medication and leave. The last thing you want is to not get your medication. So if you have to pay then you just pay.’ (NEL_M4, female, high-income corporate co-payer)

The state of mind of an ill person appears to affect their ability to actively and effectively engage with co-payments at the point of sale at pharmacies.

### Purchaser preferences: Pharmacy choice

Corporate pharmacies appear to be preferred by both medical schemes and members. Independent pharmacies were perceived to have limited stock availability compared to corporate pharmacies:

‘I have never co-paid at other pharmacies. I only co-pay at big corporate pharmacies because it is preferred by my medical scheme. The scheme told me to go there. Maybe they didn’t know that I have to co-pay there.’ (KER_ M12, female, high-income corporate co-payer)

‘I never go to one-man pharmacies for prescription medication. I always go to chain pharmacies. I assume that corporate pharmacies will always have your prescription unlike small pharmacies. Small pharmacies have limited stock capabilities and chances are high that a small pharmacy will give me an alternative medicine which was not prescribed by the doctor.’ (DUM_M8, male, high-income corporate non-co-payer)

The scheme’s motive for channelling members to pharmacies that charge co-payments might require further investigation to understand.

### Purchaser preferences: Medicine choice

The perceptions about quality of generics were mixed; however, the majority of medical scheme members believed that generics were inferior compared to originators.

‘To be quite honest, I question generics … whether they are inferior or weak or what. I’m not sure if they are of [the] same quality as originators. I think generics take much longer to work. Also, why do they make them so cheap?’ (LES_M11, male, corporate low-income co-payer)

‘My assumption is … to do a generic they tweak the process of developing the medicine. So I don’t know what reactants are used to develop this ‘same thing’. Maybe they use hydrochloric acid instead of sulphuric acid. I wouldn’t know whether there are impurities but I assume the doctor knows.’ (DUM_M8, male, high-income corporate non-co-payer)

‘The quality between generics and originator is the same except for price differences. Sometimes generics are made by the same company that makes the originator. So, there is no difference between generics and originators.’ (MAN_M6, female, high-income independent non-co-payer)

There appear to be perceptions that low medicine prices are synonymous with poor quality and that the prescribing doctor’s choice cannot be changed.

### Professionals with an influence on purchasers

When professionals shared personal experiences about medicines, patients felt assured about their effectiveness. Trust in a doctor or pharmacist appeared to be based on the perceived level of care exercised during consultation. Some accepted the doctor’s instruction out of fear even when they felt uncomfortable:

‘I was with the ophthalmologist. When I got there they told me this is the best eye drop ever, I take it too. They tell you they take it every day. So now I’m stuck with it.’ (FUT_M3, female, high-income independent co-payer)

‘I prefer to go all the way to my pharmacy where I used to stay. They care about you and you can discuss stuff with them and they even look after my mom and my dad. So I know what I would be paying for with them. I sometimes don’t even [*make a*] co-payment.’ (LES_M11, male, corporate low-income co-payer)

‘The doctor gambles with our lives but you have to accept what he says … Eish, who can argue with these doctors? [*giggling*] Sometimes I think doctors treat us like guinea pigs to check if medicines work or not. I think they are guessing sometimes, so I rely on my pharmacy for a second opinion.’ (LES_M11, male, corporate low-income corporate co-payer)

Patients appeared to be willing to spend more money if they felt the professional cared about their well-being. Trusted pharmacists were consulted immediately after consultation with the doctor who was deemed to be unconvincing or unbelievable.

### The influence of pharmacy stock and stakeholder relationships along the supply chain

The stock available at the pharmacy determined which medicine got dispensed, and pharmacists considered viability when dispensing. When the price of a purchased medicine was higher than that for which the scheme was prepared to pay, pharmacists would charge the difference as co-payment:

‘At the pharmacy, if you don’t agree to the price set by the scheme then the scheme’s client will always co-pay. Affordability and viability plays a huge role on co-payments. It is better to have lesser profit but many times [more sales] because at least you get more members coming to your pharmacy.’ (CRO_ P5, male, pharmacist at an independent pharmacy located in a high-income area)

‘We dispense from our stock, which is determined by the availability list, which is decided at head office. The list of preferred medicines is highlighted for us on the screen when we dispense. We always keep stock of medicines that appear on the availability list. We have brown lines which we don’t keep, blue lines which are mostly expensive and green lines which are preferred by business. I have noticed that our business prefers certain manufacturers. We also negotiate with manufacturers. Manufacturers sponsor us financially with staff training and conferences and you can imagine when this happens you have to be loyal to the manufacturer.’ (VER_P6, male, pharmacist at a corporate pharmacy located in a high-income area)

The profitability of the pharmacy business and relationships with pharmaceutical manufacturers are prioritised by dispensing pharmacists, and the purchaser position appears to be rarely considered.

### The effect of brand loyalty

Pharmacists suggested that familiarity with a specific brand resulted in patients being loyal to particular brands:

‘When the scheme introduces a new cheaper medicine on the formulary list, then you find patients wanting to stick to what they are used to, which they call the original. Usually the change causes a co-payment, but scheme members don’t know this logic.’ (CRO_P5, male, pharmacist at an independent pharmacy located in a high-income area)

‘When patients take medicines for a long period, brand loyalty develops and this may cause them to start co-paying, especially when medical schemes change the reimbursement criteria. If a patient feels comfortable with a medicine, they do not understand why they should be changed to another medicine. They co-pay if they don’t change.’ (KAL_P4, male, pharmacist at a corporate pharmacy located in a low-income area)

Because patients sometimes preferred to use specific medicines, these are demanded at pharmacies. In response, because pharmacists focus more on profitability they tend to avoid providing explanations about the financial advantages of changing to lower-cost treatment.

## Discussion

The results revealed that almost all interviewed medical scheme members and dispensing pharmacists, regardless of socio-economic status, were confused by co-payment practices. While medical scheme members believed themselves to have no recourse except to pay if charged a co-payment, pharmacies simply added the scheme’s unpaid shortfall amount onto the member’s invoice. The amount later gets paid OOP as a co-payment to the pharmacy. Such an arrangement results in the scheme saving money, the pharmacy generating income and the consumer being the only stakeholder in the chain remaining without much benefit in the process.^8^ The findings also revealed that among stakeholders involved in the medicine purchasing process, the medical scheme member is the only one unable to enjoy full protection of their interests.^16^

Overall, co-payments appeared to be outcomes of an interplay between a number of factors, which included the following: profit-oriented stakeholders within the pharmaceutical supply chain; relationships between some stakeholders, excluding the purchaser; negative consumer perceptions about generics and low-priced medicines; purchaser’s loyalty to certain brands of medicines; perceptions about availability of certain services and medicine stock at different types of pharmacies (corporate or chain pharmacies vs. independent pharmacies); fear of the patient to override the prescribing doctor’s choice of prescription medicine; lack of active monitoring of the use of unintelligible language during communication of financial information between medical schemes and their members; absence of incentives for pharmacies to dispense the lowest-priced medicine; and limited protection of consumers from undesirable practices and relationships between stakeholders involved in the sale of medicines. It is reassuring, however, to note that universal health coverage, to be introduced in the form of National Health Insurance in South Africa, intends to provide financial risk protection to consumers and to eliminate, among other costs, user fees such as co-payments charged by service providers at private healthcare facilities.^7^ The elimination of the fee for service model, which is currently in use in most private sector healthcare facilities, and the reintroduction of the reference price lists for services rendered, have potential to enhance efforts towards the achievement of National Health Insurance goals.

Interestingly, although co-payments were paid to pharmacists, medical scheme members were angry with medical schemes for refusing to co-pay on their behalf at pharmacies. This could be because pharmacy personnel explained co-payments to consumers in a manner that somehow rendered schemes responsible for causing co-payments rather than the pharmacies. These explanations about co-payments could even be distorted to make even pharmacies appear as victims to the decisions and rules of the schemes. Regulator views about legislative interventions and dispensing practices suggested that co-payments were caused by lack of policing and enforcement of medicine-pricing policies in South Africa. In the Netherlands, a study conducted by Wettermark et al.^17^ confirmed that most pharmaceutical policy strategies generally lacked thorough enforcement. It was interesting to note that the doctor’s instructions were taken by members out of fear of the authority of the professional rather than out of having faith in their specialty. In hindsight, the researcher could have included prescribing doctors in the study population, to establish whether the prescriber does in any way feel obliged to comply with the scheme’s reimbursement criteria and whether co-payments, paid by medical scheme members at pharmacies and formulary lists, are in some way considered prior to writing a prescription.

The sampling strategy used in this small study ensured the diversity of views about co-payments by insured patients attending retail pharmacies in the Pretoria area; however, it was a small sample. The study highlighted the need for consumers to be educated about medicine prices, particularly about information on generics. The importance of taking note of variations in prices of medicines that yield the same treatment outcome and the fact that branded generics may be far more expensive than unbranded medicines are some components of the information to be communicated regularly to consumers. A larger study would be of value to confirm these findings and to explore the contribution of prescribing doctors in the consumers’ exposure to co-payments.

## Conclusion

This study found that factors contributing to co-payments appeared to include perception-based and profit-driven decisions made by both purchasers and suppliers of pharmaceuticals. Lack of comprehension of co-payment information by medical scheme members was poor and improvements in the communication methods and channels used by medical scheme organisations are required. Active monitoring of stock at pharmacies, education about generic equivalence and price variations between similar medicines, regulation of the language used by schemes to communicate with their members and decisive introduction of already proposed National Health Insurance principles could collectively contribute towards the reduction of co-payments.
